# Kinematic and kinetic characteristics exhibit differences in two typical progressive movements of Baduangong and Tai Chi: a comparison based on personalized musculoskeletal simulation analysis

**DOI:** 10.3389/fbioe.2025.1726221

**Published:** 2026-01-12

**Authors:** Peng Yang, Boya Xiao, Canwen Liu, Qifei He, Weichao Sun, Yang Lv, Zexing Huang, Shuchai Xu, Yan Liu, Yongjin Li, Dingkun Lin, Da Guo

**Affiliations:** 1 Orthopedics Department, The Second Affiliated Hospital of Guangzhou University of Chinese Medicine, Guangzhou, Guangdong, China; 2 Guangzhou University of Chinese Medicine, Guangzhou, Guangdong, China; 3 Shenzhen Institutes of Advanced Technology, Chinese Academy of Sciences, Shenzhen, Guangdong, China; 4 Orthopedics Department, Cenxi Hospital of Chinese Medicine, Wuzhou, China; 5 Orthopedics Department, Shenzhen second people’s Hospital, Shenzhen, Guangdong, China

**Keywords:** Baduangong, gait analysis, musculoskeletal simulation, Qigong, Tai Chi

## Abstract

**Purpose:**

This study aims to analyze the lower limb kinematic and kinetic characteristics of these two Qigong progressive movements, laying a preliminary biomechanical foundation for exploring their potential applications in targeted movement-based interventions.

**Methods:**

A total of 21 non-athletic healthy students were recruited, all of whom had completed Qigong course training. Motion capture equipment was used to collect motion data and ground reaction force data of each participant, including data for 8th movement of Baduangong (BDG (8th)), warding off in advancing of Tai Chi (TC (WOA)), and normal walking gait (NG (Walk)). The AnyBody Modeling System was used for personalization to construct a musculoskeletal model based on the participants’ body weight and fat percentage. Kinematic and inverse dynamics analyses were conducted to obtain lower limb joint range of motion (ROM), joint reaction forces, and moments.

**Results:**

Two progressive movements had similar gait cycles (9–10 s), longer than NG (Walk) (1.15 ± 0.04 s, p < 0.01), with larger joint ROM (especially hip/knee flexion-extension). Their lower limb joint forces, moments were higher than NG (Walk) (p < 0.05); Comparing the two progressive movements, TC (WOA) exhibited higher peak joint reaction forces (p < 0.05); while BDG (8th) demonstrated greater peak hip abduction and extension moments (p < 0.05).

**Conclusion:**

The two progressive movements share the Qigong characteristic of “slowness and continuity”, but they exhibit different biomechanical features due to different movement design concepts. BDG (8th) is characterized by “fast rhythm, long stride length, and flexible transformation”, and exhibits high hip abduction moments. TC(WOA) is characterized by “slow rhythm, short stride length, and phased regulation” and shows kinematic features associated with multi-directional joint movement control. These findings lay a preliminary biomechanical foundation for exploring the potential differentiated applications of the two Qigong movements.

## 1 Introduction

Qigong, a traditional Chinese exercise, is widely recognized as a health-promoting form of physical activity, with Tai Chi (TC) being the most renowned. As a type of Qigong characterized by slow, continuous, and cyclic movements (Law and Li, 2022), TC has been extensively applied in disease intervention and rehabilitation over the past decades (Zhang T. et al., 2024; Liang et al., 2024; Zhang W. et al., 2024). Lin’s Baduangong (BDG), a novel Qigong discipline developed by Dingkun Lin. This exercise method has been developed by integrating and refining various Qigong and martial arts patterns, comprising 8 movements that include fundamental actions such as heel lifting, squatting, and walking. In previous clinical studies (Chen et al., 2015), we observed that BDG exerts positive and effective effects on the prevention, management, and treatment of chronic musculoskeletal disorders involving the spine and lower limb joints. While these studies have confirmed the efficacy of BDG from a clinical perspective, in-depth biomechanical research on its lower limb movement characteristics-specifically, kinematic parameters, kinetic features, and neuromuscular activation patterns of relevant muscle groups-remains lacking. This gap hinders a clear understanding of the underlying biomechanical mechanisms through which BDG exerts its therapeutic effects.

The application of 3D motion capture technology to collect human movement trajectory data and drive musculoskeletal models enables kinematic and dynamic simulation analysis of specific movements. Relevant research has been conducted on biomechanical simulations of traditional Qigong forms such as TC, which have revealed the standardization and scientificity of Qigong movements through comparative analyses of specific actions (Li et al., 2023a). For instance, TC-Bafa Wubu, a new type of simplified TC, includes five classic steps: Jin (advancing), Tui (retreating), Pan (shifting right), Gu (shifting left), and Ding (stabilizing). Several studies have conducted simulation analyses of lower limb stepping movements in TC-Bafa Wubu, elucidating distinct mechanical characteristics, such as high symmetry between the left and right limbs, larger proximal muscles contribute to movement stability and overall control, while distal muscles contribute to fine motor control and forward propulsion (Zhang X. et al., 2024; Hua et al., 2022). Notably, lower limb movements in progressive steps of TC-Bafa Wubu, such as “warding off in advancing” (TC (WOA)), share similarities with normal walking gait while exhibiting unique movement patterns, suggesting their potential value in lower limb rehabilitation training (Li et al., 2023b). BDG also incorporates diverse lower limb stepping motions, among which the 8th movement (BDG (8th)), termed “wandering step” focuses on progressive movements and exhibits distinct lower limb gait phase characteristics, including striding, supporting, and swinging. To date, no simulation analyses have compared the progressive movements of BDG and TC, nor explored their specific kinematic and mechanical features.

In this study, 3D motion capture equipment was used to collect motion data of the progressive movements of BDG (8th) and TC (WOA). Motion simulation analyses were performed using the AnyBody musculoskeletal model to compare the lower limb kinematic and biomechanical characteristics of these two progressive movements. The findings are expected to clarify the biomechanical differences between different Qigong exercises in enhancing lower limb joint, muscle, and bone functions, thereby providing a scientific basis for selecting movements that emphasize certain mechanics in rehabilitation and disease intervention.

## 2 Materials and methods

### 2.1 Participants

A total of 21 healthy, non-athletic students from Guangzhou University of Chinese Medicine were recruited for this study (none of the participants engaged in professional or competitive sports training, nor did they have a background of regular high-intensity athletic practice). Sample size was determined based on previous comparable studies investigating the biomechanics of Qigong movements, which consistently reported that a sample size of 20 participants provides adequate statistical power to detect meaningful kinematic/kinetic differences in paired comparisons (Smith et al., 2025). Qigong practice is a compulsory component of the physical education curriculum at this university, requiring students to complete training in Qigong as part of their coursework. To ensure consistent and accurate performance of the movements and to reduce variability in motion-capture data, only students who had undergone 1 year of training and passed the qualification assessment were included. Participants were required to be in good health with no history of significant lower limb injuries. All participants provided written informed consent and were fully informed of the experimental procedures and objectives. This study was reviewed and approved by the Ethics Committee of Guangdong Provincial Hospital of Chinese Medicine (The Second Affiliated Hospital of Guangzhou University of Chinese Medicine). The baseline characteristics of the participants are presented in Table 1.

**TABLE 1:** Characteristics of the participants.

Characteristics	Value
Age	21 ± 1.06 years
Sex	
Female	9
Male	12
BMI	21.7 ± 2.29 kg/m^2^
Leg length	86.5 ± 8.77 cm
Pelvis width	21.6 ± 2.18 cm
Knee width	8.3 ± 0.56 cm
Ankle width	6.2 ± 0.73 cm

Data are presented as the mean ± SD, or numbers; BMI, body mass index.

### 2.2 Instruments

Motion capture was conducted in a 5 m × 6 m × 4 m room. Twelve high-precision infrared optical motion capture devices (Mars 12H, Nokov, China) were used to collect motion data at a sampling frequency of 250–500 Hz. Two 3D force plates (P-6000, BTS, Italy) were employed to measure ground reaction forces (GRF) at a sampling frequency of 1,000 Hz. Prior to data collection, the motion capture system underwent calibration procedures: static calibration was performed using the manufacturer-provided calibration frame to verify marker detection accuracy (spatial error ≤ 0.5 mm), while dynamic calibration was completed by having a test subject perform standardized movements to confirm stable marker tracking across the capture volume. Marker post-placement verification was conducted via a 5-s static standing pose to ensure no marker misalignment.

### 2.3 Experimental protocol

**FIGURE 1:**
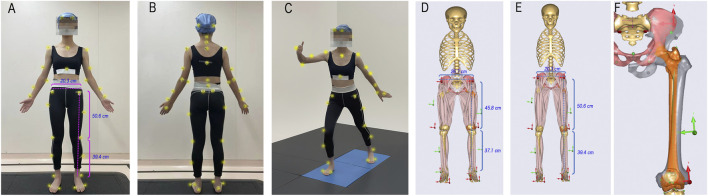
Locations of surface markers and the personalized scaled musculoskeletal model. **(A–C)** the locations of the markers placed on the subject’s body surface; the subject in the figures has a body weight of 51 kg and a height of 163 cm; **(D)** the generic lower limb musculoskeletal model; **(E)** the musculoskeletal model scaled based on body weight and fat percentage to match the subject; **(F)** the changes in bone morphology before and after scaling, where the gray part represents the bone of the generic model. During scaling, the muscle attachment points are adjusted accordingly.

Participants were instructed to wear tight-fitting clothing and were marked with standardized infrared reflective markers on their body surfaces. For accurate human simulation analysis, a total of 42 markers were placed on key anatomical landmarks of the upper and lower limbs, as well as the trunk (Figure 1).

### 2.4 Progressive movements in TC and BDG

**FIGURE 2:**
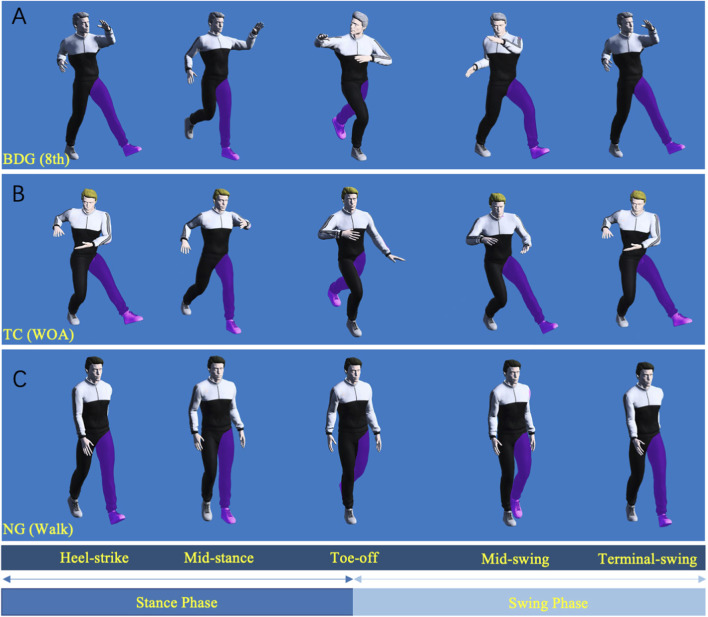
Gait phase division of the two progressive movements with reference to standardized walking gait phase classification. BDG (8th) and TC (WOA) refer to the two progressive movements; NG (Walk) refers to normal walking. **A**, BDG (8th); **B**, TC (WOA); **C**, NG (Walk).

As shown in Figure 2B, “warding off in advancing” (WOA) is a typical progressive movement in TC-Bafa Wubu. Previous simulation studies have indicated that WOA involves forces directed outward, upward, and forward from the interior (Kuo et al., 2021). The 8th movement (BDG (8th)) also representing a classic progressive movement (Figure 2A). The progressive movements of both Qigong forms exhibit distinct gait phase characteristics, sharing significant similarities with normal walking gait (Figure 2C). Therefore, this study collected three types of movements from each participant: BDG (8th), TC (WOA), and normal gait (NG (Walking)). Prior to data collection, participants performed adaptive movement trials. They were then instructed to complete a single set of movements within a specified time frame, with each foot individually stepping on the force plate to capture GRF data for the corresponding lower limb. Each movement was recorded 3 times for each participant. The order of the three movements was independently randomized for each participant to minimize potential order effects and fatigue-induced biases that might interfere with biomechanical measurements. Specifically, a random permutation of the three codes (1 = BDG (8th), 2 = TC (WOA), 3 = NG (Walking)) was generated for each participant using a Python-based program to determine their individual movement execution sequence.

To enable direct comparative analysis between the two progressive movement paradigms, the gait cycle (GC) and its constituent phases were systematically defined based on the standardized gait phase classification system (Xiong et al., 2022). A Qigong gait cycle was defined as the interval from the first ground contact of one foot to the next ground contact of the same foot. For each side, the stance phase began when the foot made ground contact and ended when the toes lifted off; the swing phase occurred between toe-off and the next ground contact. The stance and swing phases of each subject were manually segmented based on marker trajectory and GRF data. Four markers placed on the foot (including the heel and toe) were used to assess the positional relationship between the foot and the ground, while the GRF was monitored. The initial contact event was defined as when the vertical component of GRF exceeded 5% of the subject’s body weight, and the toe-off event was identified when the vertical component of GRF dropped below 5% of the subject’s body weight. Each movement was required to complete at least one full cycle for both the left and right lower limbs.

### 2.5 Data preprocessing and musculoskeletal multibody dynamic simulation

GRF curves for each gait cycle were extracted, and a clustering algorithm was used to identify characteristic curves, which were considered representative of typical movements (Simonsen, 2014). Trajectory data from these typical movements were processed and exported in C3D format for analysis using the AnyBody Modeling System (version 7.1, AnyBody Technology, Denmark), a musculoskeletal multibody dynamic simulation software. The software utilized a generic lower-limb model, which was personalized for each participant. The basic functions and applications of AnyBody technology have been well-described in previous literature (de Zee et al., 2007). Personalization involved scaling the generic lower-limb model based on individual body weight and fat percentage; the scaling formula was as follows (Equation 1):

$$F=F_{0}\frac{k_{m}}{k_{l}}\frac{R_{muscle,1}}{R_{muscle,1}},R_{muscle}=0.5-R_{fat}$$ (1)

where *F* and *F*_*0*_ are the subject musculoskeletal model maximum muscle strength; *k*_*m*_ and *k*_*l*_ are the ratio of body mass and height of the model, respectively; *R*_*muscle*_ and *R*_*fat*_ are the ratio of muscle and fat, respectively. This scaling method adjusted model mass and dimensions based on body weight and limb length, while accounting for the influence of fat percentage on bone mass, ensuring that the model’s fat percentage was consistent with the subject’s height and weight. After personalized registration, a built-in kinematic optimization algorithm was used to drive the simulation model to perform the corresponding movements. Kinematic data were collected for inverse dynamics analysis, which employed a Hill-type muscle model. This model, composed of a contractile element, a series elastic element, and a parallel elastic element, efficiently simulates human muscle properties. Model estimated muscle forces based on measured movement and body characteristics. Muscle recruitment followed the maximum-minimum recruitment auxiliary mode (Heinen et al., 2019). Inverse dynamics analysis yielded data on lower limb joint reactions, moments, and muscle forces-these muscle forces represent model estimated tension generated by muscles, rather than directly measured muscle activity, which were used for comparative analysis. To validate the accuracy of the simulation data, a GRF model was used to inversely predict lower limb GRFs, which were then compared with the experimentally measured GRF data (Daroudi et al., 2024).

### 2.6 Statistical analysis

Data were analyzed using SPSS 26.0 statistical software, and results are presented as mean ± standard deviation (mean ± SD). Prior to statistical testing, the normality of differences between paired observations was assessed using the Shapiro–Wilk test, confirming that all kinematic and kinetic variables conformed to a normal distribution. For time-series kinematic/kinetic data across movement cycles, representative summary measures were extracted to eliminate temporal correlation within cycles. These aggregated metrics included: 1. general gait parameters; 2. values of range of motion (ROM); 3. values of peak joint reaction forces; and 4. values of peak joint moments. ROM was defined as the peak-to-peak range throughout the entire movement cycle. Peak values, such as joint reaction forces and joint moments, were defined as the maximum values recorded during the movement cycle. These parameters were then subjected to paired-samples t-tests to compare differences between the two progressive movements and normal walking gait cycles. A *p* < 0.05 was considered statistically significant.

## 3 Results

**FIGURE 3:**
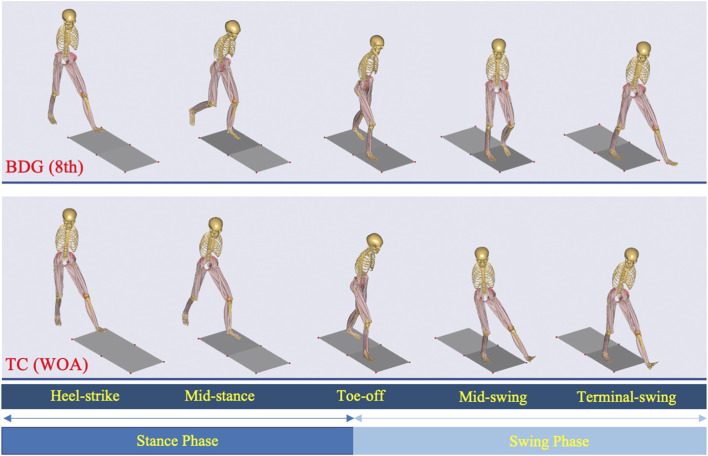
Lower-limb musculoskeletal model driven by the two progressive movements. BDG (8th) and TC (WOA) refer to the two progressive movements.

This study employed motion capture combined with musculoskeletal simulation analysis to compare the lower limb kinematic and kinetic characteristics differences between the BDG (8th) and TC (WOA), using NG (Walk) as a reference. Figure 3 shows the lower-limb musculoskeletal model driven by the movements of the two progressive movements.

### 3.1 Comparison of general gait parameters

As shown in Table 2, the gait cycle durations of the two progressive movements were similar (9–10 s), but both were significantly longer than that of NG (Walk) (1.15 ± 0.04 s), reflecting the common characteristic of Qigong as “slow and continuous”. Compared with TC (WOA), BDG (8th) exhibited significantly higher gait cadence (16.27 ± 2.54 steps/min), stride length (0.68 ± 0.05 m), and velocity (0.11 ± 0.04 m/s), while its stance phase time (7.14 ± 0.58 s), stance phase proportion (76%), and double support time (1.58 ± 0.26 s) were significantly lower (p < 0.01). There was no significant difference in step width or step length between the two progressive movements, but all parameters differed significantly from those of NG (Walk) (p < 0.01). Overall, the general gait parameters indicated that BDG (8th) was characterized by “faster rhythm, longer stride length, shorter stance phase, and higher velocity”, whereas TC (WOA) was marked by “slower rhythm, shorter stride length, longer stance phase, and lower velocity”.

**TABLE 2:** Comparison of general gait parameters.

Category	Gait cycle (s/Step)	Stance phase time (s/Step)	Stance phase proportion (%)	Stride length (m/Step)	Velocity (m/s)	Double support time (s/Step)	Cadence (Steps/min)	Step width (m/Step)	Step length (m/Step)
BDG (8th)	9.11 ± 0.92	7.14 ± 0.58	0.76 ± 0.05	0.68 ± 0.05	0.11 ± 0.04	1.58 ± 0.26	16.27 ± 2.54	0.06 ± 0.02	1.15 ± 0.03
TC (WOA)	9.74 ± 0.68	8.20 ± 0.15	0.84 ± 0.02	0.54 ± 0.11	0.05 ± 0.01	3.36 ± 0.08	12.41 ± 0.46	0.05 ± 0.02	1.21 ± 0.12
NG (Walk)	1.15 ± 0.04	0.72 ± 0.02	0.62 ± 0.04	1.24 ± 0.12	1.08 ± 0.09	0.13 ± 0.02	101.79 ± 3.57	0.02 ± 0.00	0.64 ± 0.04
*p1*	0.14	<0.01	<0.01	<0.01	<0.01	<0.01	<0.01	0.16	0.06
*p2*	<0.01	<0.01	<0.01	<0.01	<0.01	<0.01	<0.01	<0.01	<0.01
*p3*	<0.01	<0.01	<0.01	<0.01	<0.01	<0.01	<0.01	<0.01	<0.01

Data are presented as the mean ± SD. BDG (8th) and TC (WOA) refer to the two progressive movements; NG (Walk) refers to normal walking; *p1* refers to BDG (8th) vs. TC (WOA); *p2* refers to BDG (8th) vs. NG (Walk); *p3* refers to TC (WOA) vs. NG (Walk).

### 3.2 Comparison of lower limb joint kinematics

**FIGURE 4:**
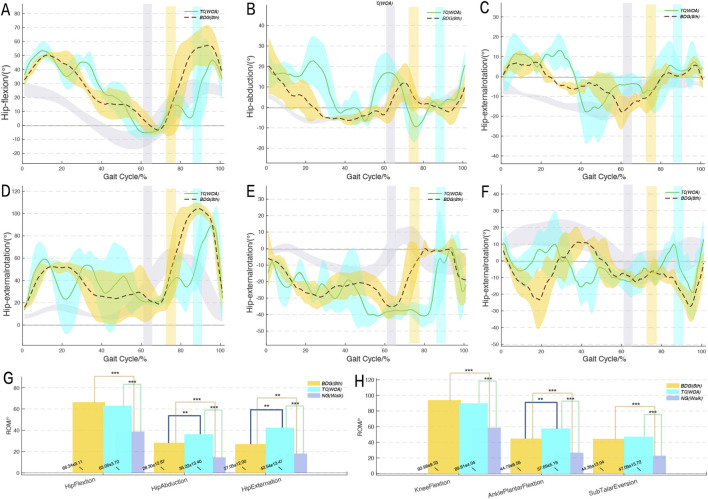
ROM trajectories of lower limb joints during two progressive movements compared to normal walking. Curves represent the mean values, shaded areas indicate the standard deviation range, and the bar marks the time of toe-off. The stance phase of each movement occurs before toe-off. *p < 0.05, **p < 0.01, ***p < 0.001. BDG (8th) and TC (WOA) refer to the two progressive movements; NG (Walk) refers to normal walking. ROM refer to range of motion. **A-F**, ROM trajectories of lower limb joints; **G-H**, bar chart of lower limb joints ROM.

Figure 4 clearly illustrates the ROM of lower limb joints throughout the cycle for the two progressive movements. Overall, the limb joint ROM of both progressive movements maintained a high degree of consistency in cyclic changes and showed a roughly similar trend to normal walking (with similar “peak-valley” fluctuations). This reflected the common fluctuation characteristics of the three and confirmed that the two progressive movements evolved from walking as their foundation. However, the joint ROM values of two Qigong movements were significantly larger compared to that of normal walking, particularly in the flexion-extension ROM of the hip and knee joints (BDG (8th): hip 66.34° ± 9.11°; TC (WOA): hip 63.06° ± 3.72°; BDG (8th): knee 93.98° ± 9.58°; TC (WOA): knee 89.81° ± 4.04°), and confirmed the advantage of Qigong in “expanding joint mobility”. Further analysis revealed that throughout the gait cycle, the joint ROM of BDG (8th) exhibited a trend-dominated, highly stable pattern with no obvious local amplitude fluctuations. In contrast, the curves of TC (WOA) showed superimposed minor oscillations (multiple peaks), with particularly significant differences during the stance phase. Additionally, TC (WOA) had significantly greater ROM in hip abduction-adduction, hip external-internal rotation, and ankle flexion-extension compared to BDG (8th) (p < 0.05).

### 3.3 Comparison of joint reaction forces and ground reaction forces

**FIGURE 5:**
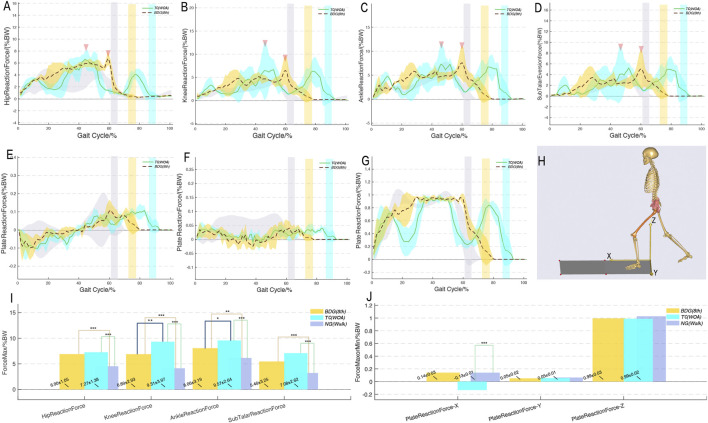
Joint reaction forces of lower limb joints and ground reaction forces trajectories during two progressive movements compared to normal walking. *p < 0.05, **p < 0.01, ***p < 0.001. BDG (8th) and TC (WOA) refer to the two progressive movements; NG (Walk) refers to normal walking. **A-G**, reaction forces trajectories; **H**, spatial localization of the musculoskeletal model with initial posture; **I-J**, bar chart of peak reaction forces.

Figure 5 shows that the peak joint reaction forces of both progressive movements were significantly higher than those of normal walking, but there was no difference in peak ground reaction forces. This suggested that Qigong stimulates joints by adjusting internal forces rather than increasing ground impact. When analyzing the joint reaction force profiles and peak forces of progressive movements, BDG (8th) exhibited a “single peak and plateau” characteristic during the stance phase: after reaching the peak in the early stance phase (20% GC), the reaction force maintained a high-level stable state until it rapidly decreased in the late stance phase (60% GC). The peak knee joint reaction force of BDG (8th) was significantly lower than that of TC (WOA) (p < 0.05). TC (WOA) showed a “multi-peak” pattern during the stance phase, with peaks appearing at 10%, 50%, and 80% GC, respectively. The gaps between peaks and valleys were obvious, and there was no obvious plateau phase, exhibiting a sharp peak-like pattern. Its peak knee and ankle joint reaction forces were significantly higher than that of BDG (8th) (p < 0.05).

### 3.4 Comparison of hip and knee joint moments

**FIGURE 6:**
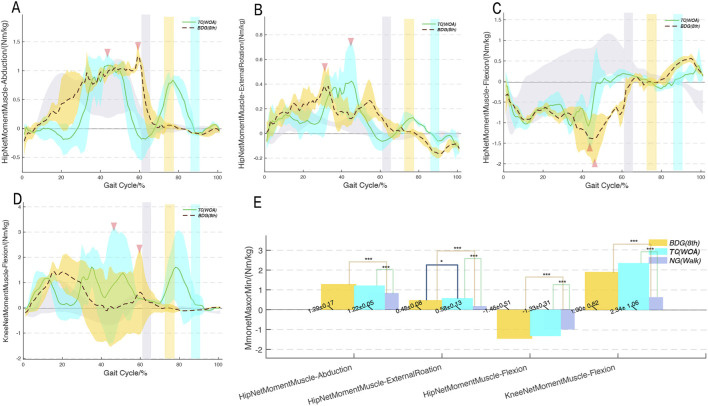
Hip and knee net joint moments trajectories during two progressive movements compared to normal walking. *p < 0.05, **p < 0.01, ***p < 0.001. BDG (8th) and TC (WOA) refer to the two progressive movements; NG (Walk) refers to normal walking. **A-D**, joint moments trajectories; **E**, bar chart of peak joint moments.

**FIGURE 7:**
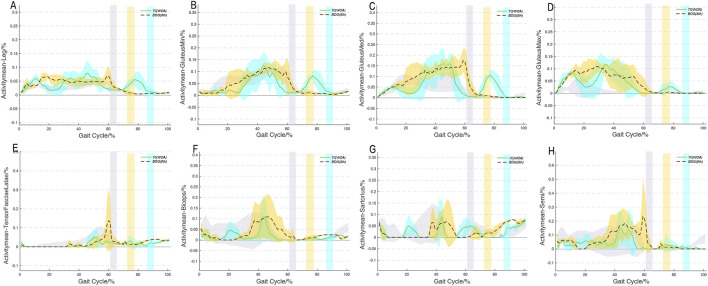
Average muscle activity trajectories (Hip abductors, extensors and Knee flexors) during two progressive movements. BDG (8th) and TC (WOA) refer to the two progressive movements. **A-H**, muscle activity trajectories.

Overall, among the three groups, the joint moment changes within the cycle showed obvious phase differences, and the peak moments of all directions in the hip and knee joints appeared during the stance phase (Figure 6). The peak hip and knee joint moments of both progressive movements were significantly higher than those of NG (Walk), with peaks occurring during the stance phase, mainly in abduction, external rotation, and extension directions (all peaks > 1 Nm/kg). The hip abduction and extension moments of BDG (8th) were significantly higher than those of TC (WOA). Figure 7 shows that, during the stance phase, the muscle activation curves of TC (WOA) exhibited greater variability, whereas those of BDG (8th) showed smaller fluctuations in activation amplitude—with this observation being particularly notable in the hip abductors.

## 4 Discussion

This study systematically compared the lower limb kinematic and kinetic characteristics of the BDG (8th) and TC(WOA) using 3D motion capture combined with AnyBody musculoskeletal simulation technology, with normal NG (Walk) as a reference. To our knowledge, it is the first study to reveal the biomechanical differences between these two progressive movements. The findings not only provide quantitative evidence for characterizing the biomechanical attributes of the two movements but also establish a preliminary biomechanical basis for exploring their potential application in rehabilitation and health promotion.

The study found that there was no significant difference in gait cycle time between BDG (8th) and TC(WOA) (both 9–10 s), but both were significantly longer than that of normal walking. This is highly consistent with the core characteristics of Qigong as “slow and continuous”. However, further parameter analysis revealed distinct gait characteristic differences between the two progressive movements. These differences mainly stem from the design philosophies of the two exercises: TC emphasizes “overcoming rigidity with flexibility and integrating movement with stillness”. Its WOA movement needs to coordinate with various complex upper limb techniques, and the slow rhythm and long stance phase help maintain the stability of the body’s center of gravity (Li et al., 2025), consistent with the biomechanical characteristics of TC as “highly symmetric and low-impact” reported in previous studies (Huang et al., 2024; Dong et al., 2022). In contrast, BDG (8th) is developed by integrating and improving multiple Qigong and martial arts patterns; its name “Youbu” (wandering steps) emphasizes the flexible movement of the lower limbs, with a focus on dynamic mobility in its striding and swinging characteristics, which aligns with the demand for “flexible shifting” in martial arts. Hence, it has a longer stride length and higher step frequency. Notably, compared with normal walking, both progressive movements showed significantly higher stance phase proportions, suggesting that Qigong movements may increase requirements for precise lower limb neuromuscular control to maintain postural stability and regulate joint mobility, which might be an important mechanism underlying their effectiveness in improving lower limb muscle strength and balance (Cui et al., 2024; Naderi and Ebrahimi, 2025). Overall, the gait characteristics of BDG (8th) are closer to normal walking, indicating that it retains the exercise-specific features while better aligning with the basic needs of daily walking.

Kinematic analysis is fundamental to understanding the nature of movements. This study found that the lower limb joints ROM of the two progressive movements maintained a high degree of consistency in cycle and showed a similar trend to normal walking, confirming their common characteristic of evolving from walking gait. However, the joints ROM of both progressive movements were significantly larger than those of normal walking, highlighting the advantage of Qigong in “expanding joint mobility”, which is consistent with previous research concluding that TC can regulate joint movements through multi-angle control (Zhu et al., 2021). Among them, the flexion-extension ROM of the hip and knee joints were the largest, this movement-specific ROM characteristics may indicate potential for targeting joint mobility through regular practice. Further analysis revealed that the joints ROM of BDG (8th) exhibited high stability, while TC (WOA) often showed superimposed minor amplitude oscillations within the main trend. This difference is closely related to movement control modes: TC (WOA) needs to coordinate with upper limb movements, and the synergistic adjustment of the trunk and lower limbs may lead to high-frequency fine-tuning of joints ROM (Yu et al., 2023); in contrast, the “Youbu” (wandering steps) of BDG (8th) emphasizes the continuity of linear movement of the lower limbs, with more flexible and continuous requirements for step joint angle transitions, resulting in more stable curves. Additionally, BDG (8th) showed significantly smaller ROM in hip abduction-adduction, hip external-internal rotation, and ankle flexion-extension compared to TC (WOA), indicating that TC (WOA) has higher demands for multi-directional joint movements of the lower limbs and may be more suitable for populations needing to improve multi-angle joint control ability. In terms of movement style, BDG (8th) achieves “faster rhythm and longer stride length” through flexible joint movements, reflecting the characteristic of “flexible transformation”; TC (WOA), characterized by multi-directional fine-tuning and slow rhythm, aligns with the style of “gentle regulation”. These differentiated features provide precise references for clinical selection.

Kinetic analysis revealed the core mechanisms by which the two progressive movements stimulate the body. The study found that the peak lower limb joint reaction forces of both exercises were significantly higher than those of normal walking, but there was no significant difference in peak ground reaction forces among the three, suggesting that Qigong movements stimulate joints by adjusting the distribution of lower limb internal forces (Wu and Millon, 2008) (rather than increasing ground impact), which is consistent with the “low-impact” property of traditional Qigong. Specifically, the peak knee and ankle joint reaction forces of TC (WOA) were significantly higher than those of BDG (8th), and the stance phase reaction forces showed a “multi-peak” characteristic, reflecting that its slow movement accompanied by multiple center-of-gravity adjustments leads to fluctuations in joint loads. In contrast, BDG (8th) showed a “single peak and plateau phase” characteristic in stance phase reaction forces (maintaining stability after peaking at 20% GC), suggesting that its continuous movements allow the center of gravity to slide smoothly, reducing the risk of joint load fluctuations. In terms of joint moments, the moments of both progressive movements were higher than those of normal walking (Chen et al., 2020). Notably, the hip abduction moment of BDG (8th) was significantly higher than that of TC (WOA), and it exhibited a characteristic plateau phase with fewer fluctuations during the stance phase. This result may suggest that compared with TC (WOA), BDG (8th) maintains body stability during the stance phase through more stable hip abduction moment output, which may be associated with the sustained activation of the hip abductor muscles (Wang and Syed Ali, 2024).

Through personalized musculoskeletal simulation (considering the influence of body weight and fat percentage on musculoskeletal parameters), this study is the first to systematically clarify the biomechanical differences between the progressive movements of BDG and TC. Although both originate from walking gait and follow basic gait mechanical laws, they have formed unique stylistic differences due to different movement design philosophies. BDG (8th) takes “fast rhythm, flexibility, and stability” as its core, with muscle activation mainly involving abductor and extensor muscle groups, showing the smaller fluctuations muscle stimulation mode, and focusing more on the continuity and coherence of lower limb movements. In contrast, TC (WOA) centers on “slow rhythm and phased regulation”, with muscle activation also mainly involving abductor and extensor muscle groups but showing “fluctuating” characteristics, and is more conducive to improving joint ROM and coordination control ability. These findings offer a preliminary scientific rationale for exploring the differentiated clinical application potential of the two progressive movements, though direct extrapolation to patient populations is limited by the exclusive inclusion of healthy young adults in the current study. For instance, BDG (8th) may hold potential value for individuals with clinical conditions that require improvements in walking efficiency and hip muscle strength (Bizzini et al., 2023; Yang et al., 2023); while TC (WOA) could be beneficial for populations where enhanced joint flexibility and balance are therapeutic priorities (Li et al., 2024; Toloraia et al., 2024). Future studies can further explore the intervention effects of the two exercises on specific diseases, combined with long-term follow-up data, to provide more in-depth guidance for the modern and precise application of Qigong.

## Data Availability

The raw data supporting the conclusions of this article will be made available by the authors, without undue reservation.

## References

[B1] Bizzini M. Schaub G. Ferrari E. Monn S. Leunig M. Casartelli N. C. (2023). Hip muscle strength in male and female patients with femoroacetabular impingement syndrome: comparison to healthy controls and athletes. Phys. Ther. Sport 61, 142–148. 10.1016/j.ptsp.2023.03.010 37054534

[B2] Chen S. Su G. Xu H. Zhao B. Hou Y. Lin D. (2015). “Baduangong exercise provides new insights into neck type of cervical spondylosis,” in Proceedings of the 2015 international conference on education, management, information and medicine.

[B3] Chen P. J. Penn I. W. Wei S. H. Chuang L. R. Sung W. H. (2020). Augmented reality-assisted training with selected Tai-Chi movements improves balance control and increases lower limb muscle strength in older adults: a prospective randomized trial. J. Exerc Sci. Fit. 18 (3), 142–147. 10.1016/j.jesf.2020.05.003 32514277 PMC7265060

[B4] Cui J. Hao Z. Tian H. Yang Y. Wang J. Lin X. (2024). The effects of Tai Chi on standing balance control in older adults may be attributed to the improvement of sensory reweighting and complexity rather than reduced sway velocity or amplitude. Front. Aging Neurosci. 16, 1330063. 10.3389/fnagi.2024.1330063 38650868 PMC11033441

[B5] Daroudi S. Arjmand N. Mohseni M. El-Rich M. Parnianpour M. (2024). Evaluation of ground reaction forces and centers of pressure predicted by AnyBody modeling system during load reaching/handling activities and effects of the prediction errors on model-estimated spinal loads. J. Biomech. 164, 111974. 10.1016/j.jbiomech.2024.111974 38331648

[B6] de Zee M. Hansen L. Wong C. Rasmussen J. Simonsen E. B. (2007). A generic detailed rigid-body lumbar spine model. J. Biomech. 40 (6), 1219–1227. 10.1016/j.jbiomech.2006.05.030 16901492

[B7] Dong X. Hu X. Chen B. (2022). Biomechanical analysis of arm manipulation in Tai chi. Comput. Intell. Neurosci. 2022, 2586716. 10.1155/2022/2586716 35755753 PMC9232327

[B8] Heinen F. Sørensen S. N. King M. Lewis M. Lund M. E. Rasmussen J. (2019). Muscle-tendon unit parameter estimation of a hill-type musculoskeletal model based on experimentally obtained subject-specific torque profiles. J. Biomech. Eng. 141 (6), 061005. 10.1115/1.4043356 30942825

[B9] Hua H. Zhu D. Wang Y. (2022). Comparative study on the joint biomechanics of different skill level practitioners in Chen-Style Tai Chi punching. Int. J. Environ. Res. Public Health 19 (10), 5915. 10.3390/ijerph19105915 35627452 PMC9141462

[B10] Huang R. Ma Y. Lin S. Zheng W. Liu L. Jia M. (2024). Correlation between the biomechanical characteristics and stability of the 143D movement during the balance phase in competitive Tai Chi. Front. Bioeng. Biotechnol. 12, 1449073. 10.3389/fbioe.2024.1449073 39444520 PMC11496090

[B11] Kuo C. C. Chen S. C. Wang J. Y. Ho T. J. Lin J. G. Lu T. W. (2021). Effects of Tai-Chi chuan practice on patterns and stability of lower limb inter-joint coordination during obstructed gait in the elderly. Front. Bioeng. Biotechnol. 9, 739722. 10.3389/fbioe.2021.739722 34993183 PMC8724780

[B12] Law N. Y. Li J. X. (2022). Biomechanics analysis of seven Tai Chi movements. Sports Med. Health Sci. 4 (4), 245–252. 10.1016/j.smhs.2022.06.002 36600972 PMC9806716

[B13] Li H. Wang X. Du Z. Shen S. (2023a). Analysis of technical characteristics of typical lower limb balance movements in Tai Chi: a cross-sectional study based on AnyBody bone muscle modeling. PeerJ 11, e15817. 10.7717/peerj.15817 37551348 PMC10404393

[B14] Li H. Peng F. Lyu S. Ji Z. Li Y. (2023b). Study on two typical progressive motions in Tai Chi (bafa wubu) promoting lower extremity exercise. Int. J. Environ. Res. Public Health 20 (3), 2264. 10.3390/ijerph20032264 36767630 PMC9915851

[B15] Li G. Huang P. Cui S. He Y. Jiang Q. Li B. (2024). Tai chi improves non-motor symptoms of parkinson's disease: one-year randomized controlled study with the investigation of mechanisms. Park. Relat. Disord. 120, 105978. 10.1016/j.parkreldis.2023.105978 38244460

[B16] Li W. Liang M. Xiang L. Radak Z. Gu Y. (2025). A cross-sectional study on the biomechanical effects of squat depth and movement speed on dynamic postural stability in Tai chi. Life (Basel) 15 (6), 977. 10.3390/life15060977 40566627 PMC12193758

[B17] Liang L. Zhang M. Li K. Hou J. Wu C. (2024). A trend of Tai chi in osteoporosis research: a bibliometric analysis. Complement. Ther. Med. 86, 103083. 10.1016/j.ctim.2024.103083 39284420

[B18] Naderi A. Ebrahimi S. Z. (2025). Effects of Tai chi training on functionality, dynamic balance, kinesiophobia, and quality of life in athletes with functional ankle instability. Res. Sports Med. 33 (1), 48–61. 10.1080/15438627.2024.2387350 39099186

[B19] Simonsen E. B. (2014). Contributions to the understanding of gait control. Dan. Med. J. 61 (4), B4823. 24814597

[B20] Smith J. Jackson T. Liu W. Gelfond J. Hsiao H. Y. (2025). Combined effects of Tai-Chi gait with mediolateral ground support perturbation on dynamic balance control. Sports Med. Health Sci. 7 (3), 208–213. 10.1016/j.smhs.2024.07.002 39991122 PMC11846441

[B21] Toloraia K. Gschwandtner U. Fuhr P. (2024). High-frequency multimodal training with a focus on Tai Chi in people with parkinson's disease: a pilot study. Front. Aging Neurosci. 16, 1335951. 10.3389/fnagi.2024.1335951 38425785 PMC10902121

[B22] Wang F. Syed Ali S. K. B. (2024). Health benefits of short Taichi qigong exercise (STQE) to university students' core strength, lower limb explosive force, cardiopulmonary endurance, and anxiety: a quasi experiment research. Med. Baltim. 103 (13), e37566. 10.1097/MD.0000000000037566 38552100 PMC10977524

[B23] Wu G. Millon D. (2008). Joint kinetics during Tai chi gait and normal walking gait in young and elderly Tai chi chuan practitioners. Clin. Biomech. (Bristol) 23 (6), 787–795. 10.1016/j.clinbiomech.2008.02.001 18342415

[B24] Xiong B. Yang P. Lin T. Xu J. Xie Y. Guo Y. (2022). Changes in hip joint contact stress during a gait cycle based on the individualized modeling method of “gait-musculoskeletal system-finite element”. J. Orthop. Surg. Res. 17 (1), 267. 10.1186/s13018-022-03094-5 35568957 PMC9107226

[B25] Yang P. Liu Q. Lin T. Aikebaier A. Jiang L. Sun W. (2023). Mechanical upside of PAO mainstream fixations: co-simulation based on early postoperative gait characteristics of DDH patients. Front. Bioeng. Biotechnol. 11, 1171040. 10.3389/fbioe.2023.1171040 37539435 PMC10396769

[B26] Yu H. Wang J. Mao M. Song Q. Zhang C. Fong D. T. P. (2023). Muscle co-contraction and pre-activation in knee and ankle joint during a typical Tai chi brush-knee twist-step. Res. Sports Med. 31 (5), 628–637. 10.1080/15438627.2021.2020788 34957881

[B27] Zhang T. Li L. Hondzinski J. M. Mao M. Sun W. Song Q. (2024a). Tai chi counteracts age-related somatosensation and postural control declines among older adults. J. Exerc Sci. Fit. 22 (2), 152–158. 10.1016/j.jesf.2024.02.004 38444520 PMC10912684

[B28] Zhang W. Wang H. Xiong Z. Li C. (2024b). Efficacy of Tai Chi exercise in patients with hypertension: systematic review and meta-analysis. Curr. Probl. Cardiol. 49 (11), 102798. 10.1016/j.cpcardiol.2024.102798 39208601

[B29] Zhang X. Jia M. Ke Y. Zhou J. (2024c). Neuromuscular synergy characteristics of Tai Chi leg stirrup movements: optimal coordination patterns throughout various phases. Front. Bioeng. Biotechnol. 12, 1482793. 10.3389/fbioe.2024.1482793 39506976 PMC11538057

[B30] Zhu Q. Zhou X. Zhang S. Fang M. Li J. X. (2021). Joint angles and joint moments of the lower limbs in four typical Tai chi movements: consideration for management of knee osteoarthritis. Res. Sports Med. 29 (6), 586–592. 10.1080/15438627.2021.1975118 34477036

