# Correction: Kinematic and kinetic characteristics exhibit differences in two typical progressive movements of Baduangong and Tai Chi: a comparison based on personalized musculoskeletal simulation analysis

**DOI:** 10.3389/fbioe.2026.1890432

**Published:** 2026-06-12

**Authors:** Peng Yang, Boya Xiao, Canwen Liu, Qifei He, Weichao Sun, Yang Lv, Zexing Huang, Shuchai Xu, Yan Liu, Yongjin Li, Dingkun Lin, Da Guo

**Affiliations:** 1 Orthopedics Department, The Second Affiliated Hospital of Guangzhou University of Chinese Medicine, Guangzhou, Guangdong, China; 2 Guangzhou University of Chinese Medicine, Guangzhou, Guangdong, China; 3 Shenzhen Institutes of Advanced Technology, Chinese Academy of Sciences, Shenzhen, Guangdong, China; 4 Orthopedics Department, Cenxi Hospital of Chinese Medicine, Wuzhou, China; 5 Orthopedics Department, Shenzhen Second People’s Hospital, Shenzhen, Guangdong, China

**Keywords:** Baduangong, gait analysis, musculoskeletal simulation, Qigong, Tai Chi

In the published article, there was a mistake in the Funding statement. The grant number for the Science and Technology Program of Guangzhou was displayed as “SL2024A04J01702”. The correct statement is “Science and Technology Program of Guangzhou (No. 2025A04J4350)”.

The original article has been updated.

